# Supplementary material for: The interactome of intact mitochondria by cross-linking mass spectrometry provides evidence for coexisting respiratory supercomplexes
Source: Mol Cell Proteomics. 2017 Dec 8;17(2):216–32. doi: 10.1074/mcp.RA117.000470 (PMC5795388; doi:10.1074/mcp.RA117.000470)
Supplement: Supplemental Data [file supp_RA117.000470_133922_0_supp_23978_fzffwf.zip › spectra_annotation/mito_spectra_annotation/445-1-8-1-16-1.pdf]

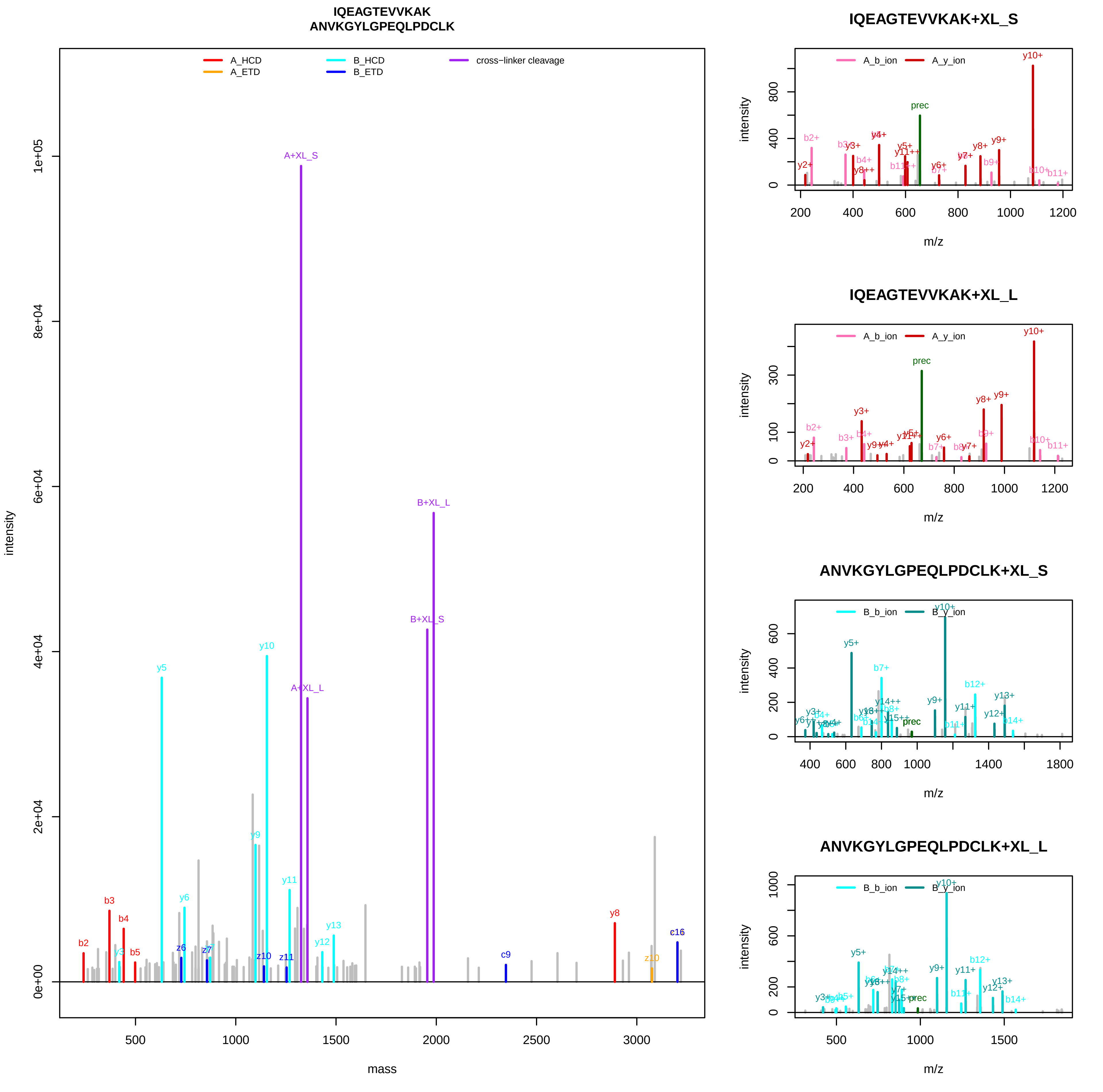
IQEAGTEVVKAK
ANVKGYLGPEQLPDCLK
A_HCD
A_ETD
B_HCD
B_ETD
cross−linker cleavage
A+XL_S
B+XL_L
B+XL_S
A+XL_L
b2
b3
b4
b5
y3
y5
y6
z6
z7
y9
y10
z10
y11
z11
y12
y13
c9
y8
z10
c16
intensity
mass
0e+00
2e+04
4e+04
6e+04
8e+04
1e+05
500
1000
1500
2000
2500
3000
IQEAGTEVVKAK+XL_S
A_b_ion
A_y_ion
prec
y10+
b2+
y2+
b3+
y3+
y4+
b4+
y8++
y5+
y11++
b11++
y6+
b7+
y7+
y8+
y9+
b9+
b10+
b11+
intensity
m/z
IQEAGTEVVKAK+XL_L
ANVKGYLGPEQLPDCLK+XL_S
B_b_ion
B_y_ion
y5+
b7+
y9+
y11+
b12+
y12+
y13+
b14+
y14++
ANVKGYLGPEQLPDCLK+XL_L
